# Prospective, Real-time Metagenomic Sequencing During Norovirus Outbreak Reveals Discrete Transmission Clusters

**DOI:** 10.1093/cid/ciy1020

**Published:** 2018-12-04

**Authors:** Amanda M Casto, Amanda L Adler, Negar Makhsous, Kristen Crawford, Xuan Qin, Jane M Kuypers, Meei-Li Huang, Danielle M Zerr, Alexander L Greninger

**Affiliations:** 1 Department of Medicine, University of Washington, Seattle; 2 Seattle Children’s Hospital, University of Washington, Seattle; 3 Department of Laboratory Medicine, University of Washington, Seattle; 4 Department of Pediatrics, University of Washington, Seattle

**Keywords:** norovirus, hospital epidemiology, outbreak, metagenomic next generation sequencing, infection prevention

## Abstract

**Background:**

Norovirus outbreaks in hospital settings are a common challenge for infection prevention teams. Given the high burden of norovirus in most communities, it can be difficult to distinguish between ongoing in-hospital transmission of the virus and new introductions from the community, and it is challenging to understand the long-term impacts of outbreak-associated viruses within medical systems using traditional epidemiological approaches alone.

**Methods:**

Real-time metagenomic sequencing during an ongoing norovirus outbreak associated with a retrospective cohort study.

**Results:**

We describe a hospital-associated norovirus outbreak that affected 13 patients over a 27-day period in a large, tertiary, pediatric hospital. The outbreak was chronologically associated with a spike in self-reported gastrointestinal symptoms among staff. Real-time metagenomic next-generation sequencing (mNGS) of norovirus genomes demonstrated that 10 chronologically overlapping, hospital-acquired norovirus cases were partitioned into 3 discrete transmission clusters. Sequencing data also revealed close genetic relationships between some hospital-acquired and some community-acquired cases. Finally, this data was used to demonstrate chronic viral shedding by an immunocompromised, hospital-acquired case patient. An analysis of serial samples from this patient provided novel insights into the evolution of norovirus within an immunocompromised host.

**Conclusions:**

This study documents one of the first applications of real-time mNGS during a hospital-associated viral outbreak. Given its demonstrated ability to detect transmission patterns within outbreaks and elucidate the long-term impacts of outbreak-associated viral strains on patients and medical systems, mNGS constitutes a powerful resource to help infection control teams understand, prevent, and respond to viral outbreaks.

## Teaser

Real-time metagenomic sequencing performed during a hospital-associated norovirus outbreak identified genetically distinct, chronologically overlapping case clusters. After the epidemiologically defined outbreak ended, ongoing transmission and shedding of the outbreak-associated virus was detected. These findings illustrate the value of genomics as a tool for infection control.

Norovirus is the most common cause of acute gastroenteritis in the United States, with an estimated incidence of 19–21 million cases per year [1]. The high prevalence of norovirus complicates efforts to prevent its introduction into health-care settings from the community [2]. Transmission primarily occurs through the fecal-oral route, though it can also occur via aerosolized viral particles and environmental contamination [3, 4]. Most cases of norovirus resolve in 12–60 hours, but hospital-associated outbreaks frequently involve immunocompromised patients, who can experience prolonged symptoms and viral shedding [5–8], and potentially act as reservoirs for viral transmission.

Metagenomic next-generation sequencing (mNGS) is increasingly used for the detection of infectious organisms when other clinical testing has been non-diagnostic. The same technique can be employed to elucidate pathogen transmission chains [9–11]. We and others have previously used real-time metagenomics to both rule in and rule out the ongoing transmission of hospital-acquired respiratory virus infections [12–14].

Here, we describe a hospital-associated outbreak of norovirus that affected 13 patients over 27 days in a large, pediatric hospital and was temporally associated with a spike in self-reported gastrointestinal symptoms among staff. We discuss the use of real-time mNGS to identify transmission clusters within the outbreak and to elucidate the long-term impacts of the outbreak, including both ongoing transmission of an outbreak strain after hospital-acquired cases had ceased and chronic shedding of hospital-acquired virus in an immunocompromised host.

## METHODS

### Setting

Seattle Children’s Hospital (SCH) is a 354-bed, tertiary-care, pediatric facility located in Seattle, Washington. The medical unit is a 64-bed unit, located on 2 floors, that cares for patients with a wide variety of acute and chronic health issues. The cancer care unit is a 48-bed unit, located on 2 floors, that cares for patients with hematologic and oncologic diagnoses, including those undergoing hematopoietic cell transplants (HCTs). These 2 units are in the same building of the hospital. All rooms on these units are private rooms with en-suite restrooms.

### Patient and Staff Case Definitions

Hospital-acquired norovirus cases were hospitalized patients who developed acute gastrointestinal symptoms (vomiting, abdominal pain, diarrhea, or nausea) more than 48 hours after admission or within 24 hours of discharge and had a positive norovirus reverse transcription polymerase chain reaction test [15] (see Supplementary Methods). Staff cases included any hospital staff member with self-reported, sudden-onset gastrointestinal symptoms.

### Case Detection

In January 2017, a routine, daily safety briefing revealed that 7 staff members assigned to the medical unit had called in sick due to gastroenteritis symptoms within a 3-day period. During initial discussions with unit staff, 4 patients were also identified with hospital-acquired gastroenteritis, with similar dates of symptom onset. Stool real-time polymerase chain reaction testing confirmed that all 4 patients had norovirus. The subsequent investigation found delays in initiating isolation precautions and revealed interactions between some ill patients and staff members. It was ultimately discovered that a recent change in the name of the norovirus test resulted in the exclusion of norovirus results from the electronic report used by the Infection Prevention Department to perform routine hospital surveillance. Therefore, a list of patients with norovirus-positive testing was obtained from the clinical laboratory, and a retrospective review was performed. This review identified 2 additional hospital-acquired norovirus cases on the cancer care unit, one occurring 4 days prior to the first case on the medical unit and one occurring 7 days prior. The first of these cases was considered the start of the outbreak (Day 0). A communication was sent to providers and nurses, requesting that patients with hospital-acquired gastroenteritis be tested for norovirus. The occupational health department monitored staff members with gastroenteritis symptoms and enforced work restrictions. By Day 33 (3 incubation periods since the last case), no further hospital-acquired cases were identified and the outbreak was considered resolved. Measures performed to control the outbreak are described in the Supplementary Methods.

### Metagenomic Next-generation Sequencing Library Generation and Sequencing

Prospective mNGS, aimed at whole-genome recovery, was performed on Days 9 and 26 of the outbreak. We sequenced samples from the hospital-acquired norovirus cases and from staff cases who tested positive for norovirus. To understand the diversity of strains circulating in the community, samples collected from patients at facilities within the University of Washington medical system, including SCH patients with community-acquired norovirus, were also sequenced (Supplementary Table S1). The mNGS sequencing was performed as described previously [12, 16] (see Supplementary Methods).

### Genotype Assignment

**Table 1.** Reference Sequence for Genotypes

ORF1: GII. P16, ORF2: GII.4, Sydney_2012 (“Sydney” genotype)	LC175468 [20]
ORF1: GII.P16, ORF2: GII.2 (“GII.2” genotype)	KY905336
ORF1: GII.P12, ORF2: GII.3 (“GII.3” genotype)	KY905334
ORF1: GII.P7, ORF2: GII.6 (“GII.6” genotype)	KU935739

Raw sequencing reads were trimmed using cutadapt and de novo assembled using SPAdes v3.11 [17, 18]. The resulting scaffolds were used as input for the online norovirus genotyping tool [19]. There were representatives of 4 genotypes among our sequences. A reference sequence was selected from GenBank for each of these genotypes (Table 1).

### Phylogenetic and Evolutionary Analysis

Consensus sequences were generated for each sequenced sample by mapping reads to the corresponding reference sequence using Geneious v10 [21]. For each sequence, single nucleotide variants (SNVs) relative to the reference were called by manual review (see Supplementary Methods). Sequences were aligned using MAFFT v7.222, with the default settings [22]. Phylogenic trees were generated using MrBayes, with chain length 1 100 000 [23]. We also determined the ratio of non-synonymous to synonymous nucleotide changes (dN/dS) over time in serial samples from Case 2.

### Sequence Clusters

We grouped 2 sequences as a cluster if they had 10 or fewer SNV differences. A sequence would be added to an existing cluster if it differed from at least 1 sequence in the cluster by 10 SNVs or less. The selection of 10 differences to define a cluster was based on past estimations of the mean norovirus genome evolutionary rate, corresponding to a time to most recent common ancestor of approximately 6 weeks, and on the distribution of pairwise differences between the sequences (see Supplementary Methods and Supplementary Figure S1).

## RESULTS

### Outbreak Characterized by Chronologically Overlapping Patient and Staff Cases

**Figure 1.**
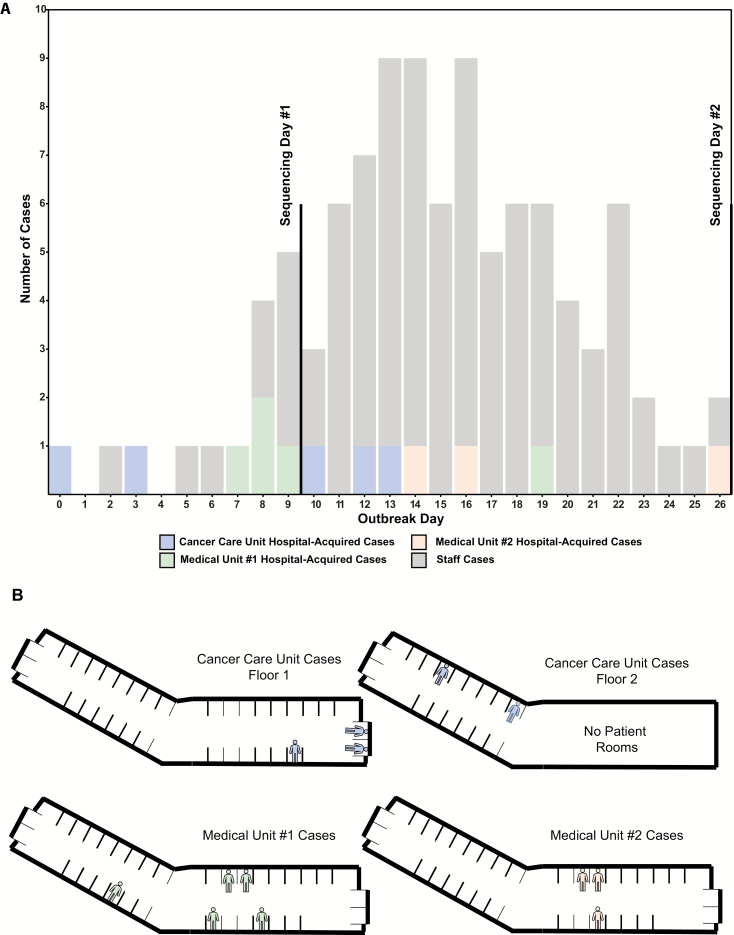
*A*, Epidemiologic curve, including both hospital-acquired norovirus cases by hospital unit and staff cases of gastrointestinal symptoms. *B*, Diagram showing location of hospital-acquired norovirus cases within the hospital.

Over a period of 27 days, there were 13 confirmed, hospital-acquired norovirus cases (Figure 1). Of these, 8 were on the medical unit and 5 were on the cancer care unit (Supplementary Notes S1 and S2). Over the same time period, 86 staff members self-reported gastrointestinal symptoms to occupational health, though only 16 (19%) of these individuals were known to be assigned to one of the affected units (9 to medical and 7 to cancer care). Norovirus testing was performed on only 2 staff members, and both were confirmed to be norovirus-positive.

### Hospital-acquired Cases Phylogenetically Cluster According to Hospital Geography

**Figure 2.**
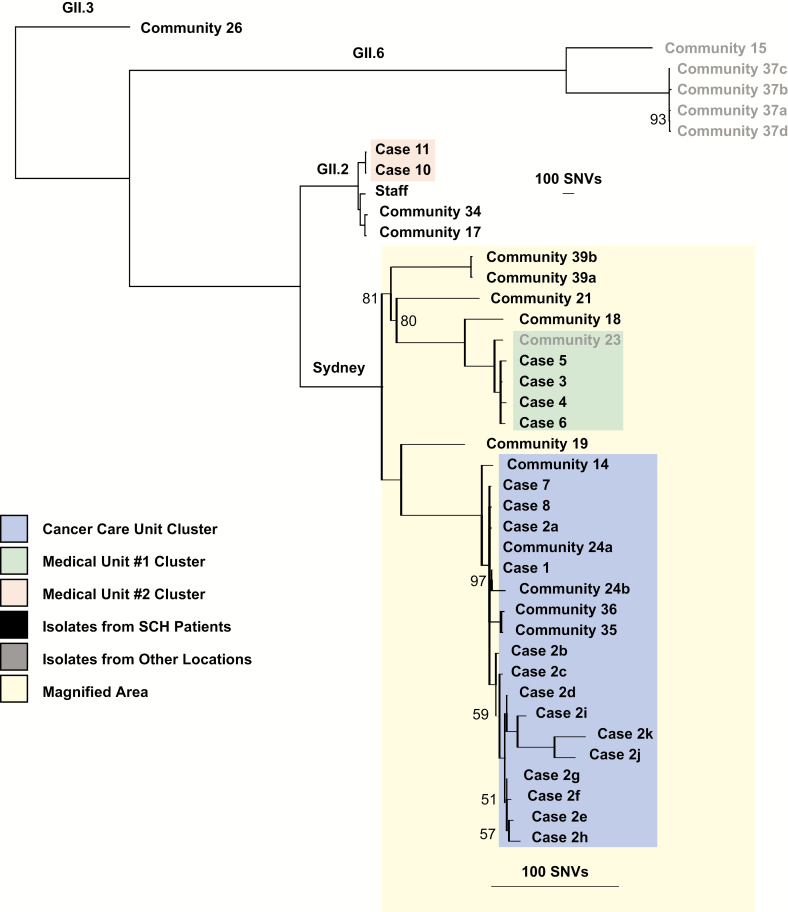
Phylogenetic tree of hospital- and community-acquired norovirus sequences. Colored boxes represent hospital-acquired case clusters. Black lettering represents samples collected at SCH, while gray lettering represents samples collected at other facilities. All non-100% posterior probabilities are denoted. Abbreviations: SCH, Seattle Children’s Hospital; SNV, single-nucleotide variants.

**Figure 3.**
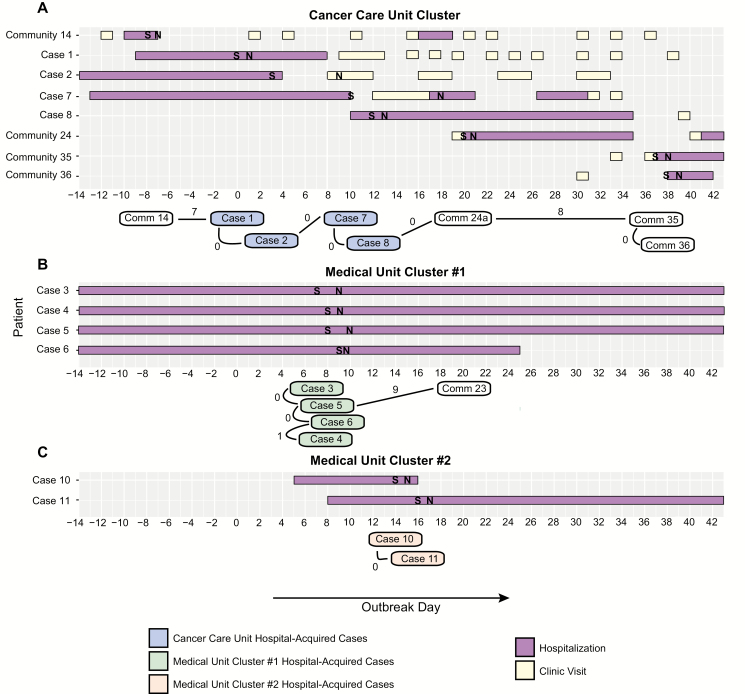
The 3 transmission clusters within the norovirus outbreak. Timelines of gastrointestinal symptoms (S), norovirus testing (N), hospitalizations, and outpatient visits for pediatric cluster members are shown on the top of each panel. The number of single-nucleotide variant differences between cluster members is shown on the bottom of each panel.

We were able to sequence samples for 10 of the 13 patient cases: 6 on the first day of prospective sequencing (Day 9) and 4 on the second (Day 26). Phylogenetic analyses of these sequences revealed 3 distinct clusters, which corresponded to hospital geography, with 1 on the cancer care unit (Cancer Care Unit Cluster) and 1 each on 2 different floors of the medical unit (Medical Unit Clusters #1 and #2; Figures 2 and 3). The 6 samples sequenced early in the outbreak were divided between the Cancer Care Unit Cluster and Medical Unit Cluster #1 (Supplementary Figure S2) and were all members of the Sydney genotype. The 4 samples sequenced later in the outbreak belonged to the Cancer Care Unit Cluster and Medical Unit Cluster #2 (Supplementary Figure S2). Samples in the Medical Unit Cluster #2 were of the GII.2 genotype. Interestingly, 4 samples from the Cancer Care Unit Cluster were genetically identical, even though they were collected up to 18 days apart (Supplementary Table S2).

On the second day of sequencing (Day 26), we were also able to generate a sequence for 1 of the 2 staff members with confirmed norovirus. This sample represented the GII.2 genotype, but was quite distinct from the 2 GII.2 patient cases (117 and 118 SNV differences).

For each of the 2 rounds of sequencing, our turnaround time from samples to phylogenetic analysis was approximately 24 hours, with information about the genetic relationships among the hospital-acquired patient cases and staff cases relayed to the infection prevention team in real time.

### Multiple Putative Cryptic Transmissions Noted Between Community-acquired and Hospital-acquired Cases

We also generated sequences for a total of 19 community-acquired norovirus infections in SCH patients and patients at other University of Washington medical facilities that were collected during and in the months after the outbreak. These samples represented 4 different norovirus genotypes. Most were genetically distinct from each other and from the 3 hospital-associated transmission clusters. There were, however, a total of 4 community-acquired infections in SCH patients that grouped with the Cancer Care Unit Cluster (Supplementary Figure S2). Of these, 1 sample was from a patient diagnosed just prior to the outbreak; the remaining 3 infections were diagnosed after the outbreak. All 4 infections were in oncology patients who were found to have shared clinic or housing space with hospital-associated case patients (Figure 3). Interestingly, a sample from an adult patient who was hospitalized at another facility during the outbreak fell within Medical Unit Cluster #1.

### Hospital-acquired Cases Were Often Persistently Positive on Follow-up Testing

Subsequent norovirus testing was performed on 6 of the 13 (46%) case patients in the 9 months following the outbreak, and 5 (83%) remained positive. These included 2 patients from the medical unit (Cases 3 and 4) and 3 patients from the cancer care unit (Cases 2, 7, and 9). Positive follow-up samples were collected a median of 57 days after the respective outbreak sample (range, 9–258 days).

### Follow-up Testing Reveals Long-term Shedding in an Immunocompromised Patient

The second hospital-acquired norovirus case (Case 2) was a 2-year-old who had received an HCT for hemophagocytic lymphohistiocyotosis (HLH), which was complicated by graft versus host disease. The patient had a total of 8 samples collected during and in the first 5 months after the outbreak (Case 2a-h). The patient’s blood cell counts were stable, and immunosuppressive medications were not significantly changed during this time. The patient then experienced reactivation of HLH and was treated with 3 months of chemotherapy, during which sample Case 2i was collected. Finally, the patient underwent a second HCT, after which samples Case 2j and Case 2k were collected. We were able to generate sequences for all 11 samples collected from this patient.

All Case 2 samples were of the same genotype, and the genetic distance between samples gradually increased with time between sample collection, suggesting chronic infection rather than resolution and re-infection. Over a period of 258 days, this patient’s norovirus accrued 65 consensus sequence changes relative to the first sample, with approximately equal numbers of synonymous (33) and non-synonymous (32) changes (Supplementary Figures S3 and S4; Supplementary Table S3). The number of non-synonymous changes was greater than the number of synonymous changes in all samples except for the last, Case 2k. The rate of accumulation of consensus changes seems to have increased slightly after the patient experienced recurrence of the HLH, received chemotherapy, and underwent a second HCT.

A number of non-synonymous changes were noted in the hypervariable or P2 subdomain of the capsid protein, which is encoded by ORF2. The P2 subdomain encodes the most immunogenic part of the capsid and amino acid substitutions here are thought to be important in immunologic escape [24]. The average value of the ratio of non-synonymous to synonymous changes (dN/dS) (when Case 2b-k were compared to Case 2a) for ORF2 was 1.717 (standard deviation 0.394). The patient’s ORF2 dN/dS was substantially larger than a previous dN/dS estimate of 0.12 for GII.4 ORF2 sequences [25]. We were unable to calculate the dN/dS ratio for the P2 subdomain, as no synonymous consensus changes were observed in this region for any of the later samples, relative to Case 2a. The number of non-synonymous consensus changes observed ranged from 1 to 8. The average mutation rate in ORF2 for the same comparisons was 0.015 substitutions/site/year (standard deviation 0.011), which again was substantially higher than GII.4 ORF2 mutation rate estimates of 5.4 × 10^–3^ substitutions/site/year [25].

## DISCUSSION

We describe a complex, hospital-associated norovirus outbreak, analyzed with prospective and retrospective mNGS, with a sample-to–phylogenetic analysis turnaround time of 24 hours for prospective sequencing.

Through the application of genomic analyses to this outbreak, we gained new insights into viral transmission among case patients and into the relationship between hospital-acquired and community-acquired cases. Firstly, we showed that what appeared to be 1 large, hospital-wide norovirus outbreak was composed of 3 genetically distinct transmission clusters, implying the introduction of 3 different norovirus strains into the hospital within a short period of time. This finding highlights the importance of health-care workers staying home when ill and of screening hospital visitors for symptoms of viral illness. Transmission of these strains within the hospital demonstrates the importance of hand hygiene, environmental cleaning, and prompt isolation of symptomatic patients, which can be challenging in patient populations that have many potential, noninfectious etiologies for vomiting and diarrhea.

Secondly, we found that there were no SNV differences among cases on the cancer care unit, suggesting to us that a contaminated fomite may have propagated the outbreak on this unit, rather than person-to-person transmission. While environmental surfaces were cleaned with bleach during the outbreak, this finding suggests that efforts should have been intensified on this unit.

Our analysis also showed a close relationship between the cancer care unit cases and several community-acquired cases in other pediatric oncology patients. This finding suggests that the cancer care unit viral strain may have continued to be transmitted after the epidemiologically defined end of the outbreak. A possible venue for these putative transmission events was the outpatient oncology clinic, highlighting the potential for viral spread in ambulatory settings, which is a particular concern for immunocompromised patients.

Finally, we showed that the noroviruses responsible for the outbreak were reflective, genetically, of the noroviruses circulating in the community at the time. In particular, both of the genotypes observed among hospital-acquired cases were seen in community-acquired cases. More broadly, our genetic analysis illustrates how outbreaks are dependent on pathogen circulation in the community. The existence of 3 discrete transmission clusters in this outbreak required that there was genetic diversity among viruses in the community, and was also likely reflective of a high overall burden of norovirus locally at the time of the outbreak, which took place in January, the typical peak of norovirus season [26].

The above findings demonstrate the utility of genetic analyses in outbreak investigations. Indeed, some of these findings would not have been apparent without the single nucleotide resolution provided by whole-genome sequencing. We have shown that mNGS can be performed in a timely manner, comparable to genotyping assays. In addition to the high-resolution genomic data they produce, mNGS approaches have the advantage of being easy to adapt for other organisms, including those for which little genomic information is available. Furthermore, data from mNGS analyses can be directly compared to data from other studies and can be pooled across studies for new analyses.

Our genetic analysis of this outbreak also gave us the opportunity to examine norovirus evolution in an immunocompromised patient who was among the hospital-acquired cases. We found that this patient’s virus accumulated non-synonymous mutations in ORF2, and particularly in the P2 subdomain, at a faster rate than synonymous mutations. This same pattern was found when examining genomes from other chronically infected, immunocompromised patients, but was not seen when the evolution of GII.4 genotype viruses at large was examined (Supplementary Note S3; Supplementary Table S4) [25]. We hypothesize that the norovirus population size in immunocompromised patients tends to be larger due to their weakened immunity. Population size is also likely more stable than in immunocompetent individuals, as a norovirus that “resides” in an immunocompromised host does not experience the bottlenecks associated with transmission from host to host. The net effects of these differences in population dynamics are that genetic drift, which can drive the fixation of neutral synonymous mutations, is weaker and that positive selection is more effective (as advantageous, non-synonymous mutations are unlikely to be lost due to drift) in immunocompromised hosts, even in the context of weakened selective pressures from the immune system. This is a possible explanation for both the surplus of non synonymous mutations and the deficit of synonymous mutations that we observed in the ORF2 gene and P2 subdomain in samples from the Case 2 patient.

In some other viral species, viruses that evolve in immunocompromised hosts seem to predict broader evolutionary trends [27]. It is currently unknown whether noroviruses from immunocompromised hosts influence viral evolution, or even if viral transmission from chronically infected patients is possible. It seems unlikely, though, that viruses from immunocompromised hosts have no impact on norovirus evolution, given that there are many degrees of immunocompromise, that the number of immunocompromised individuals continues to increase, and that immunocompromised patients have frequent contact with the health-care system and each other.

This study was limited by our inability to obtain specimens from ill staff, as most otherwise-healthy patients do not seek medical attention for norovirus infection and hospital staff often seek care from outside providers. A further limitation was the study’s mostly retrospective nature. While the use of Illumina sequencing allows for high-quality deep sequencing, the cost of reagent cartridges forces labs to use them in batched sequencing processes, which somewhat limited our ability to perform a true “real-time” analysis [28, 29]. Finally, our failure to recover genomes from some samples illustrates the challenges of working with clinical samples, as we saw variable viral RNA quality and quantity using mNGS, as compared with other methods, such as capture sequencing, that require longer turnaround times [30, 31]. Stool is a particularly daunting sample source, given the number and diversity of other organisms present [32].

Overall, our results demonstrate how metagenomics methods can be employed in outbreak investigations to elucidate intricate details about pathogen transmission. This information is vital to efforts aimed at optimizing infection-control interventions [26]. Our findings underscore the critical importance of these interventions, in both inpatient and ambulatory settings, to prevent the transmission of pathogens in health-care facilities. Infectious control efforts are perhaps particularly crucial for norovirus, a common and highly contagious organism that can have lasting impacts on medical systems and on vulnerable patient populations [33].

## Supplementary Data

Supplementary materials are available at *Clinical Infectious Diseases* online. Consisting of data provided by the authors to benefit the reader, the posted materials are not copyedited and are the sole responsibility of the authors, so questions or comments should be addressed to the corresponding author.

ciy1020_suppl_Supplementary_Figure_S1

ciy1020_suppl_Supplementary_Figure_S2

ciy1020_suppl_Supplementary_Figure_S3

ciy1020_suppl_Supplementary_Figure_S4

ciy1020_suppl_Supplementary_Tables

ciy1020_suppl_Supplementary_Notes

## Notes

***Acknowledgments.***The authors thank the patients and staff of Seattle Children’s Hospital and the University of Washington Clinical Virology Laboratory for their contributions to the outbreak response and clinical testing described here.

***Financial support.***This work was supported by departmental start-up funds from the Department of Laboratory Medicine at the University of Washington.

***Potential conflicts of interest.***A. L. G. reports personal fees from Abbott Molecular, outside the submitted work. All other authors report no potential conflicts. All authors have submitted the ICMJE Form for Disclosure of Potential Conflicts of Interest. Conflicts that the editors consider relevant to the content of the manuscript have been disclosed.

## References

[1] Hall AJ, Lopman BA, Payne DC, et al. Norovirus disease in the United States. Emerg Infect Dis 2013; 19:1198–205. 23876403 10.3201/eid1908.130465 PMC3739528

[2] Teunis PF, Moe CL, Liu P, et al. Norwalk virus: how infectious is it? J Med Virol 2008; 80:1468–76. 18551613 10.1002/jmv.21237

[3] Morter S, Bennet G, Fish J, et al. Norovirus in the hospital setting: virus introduction and spread within the hospital environment. J Hosp Infect 2011; 77:106–12. 21167622 10.1016/j.jhin.2010.09.035

[4] Repp KK, Keene WE A point-source norovirus outbreak caused by exposure to fomites. J Infect Dis 2012; 205:1639–41. 22573873 10.1093/infdis/jis250 PMC3415849

[5] Ludwig A, Adams O, Laws HJ, Schroten H, Tenenbaum T Quantitative detection of norovirus excretion in pediatric patients with cancer and prolonged gastroenteritis and shedding of norovirus. J Med Virol 2008; 80:1461–7. 18551595 10.1002/jmv.21217

[6] Sheahan A, Copeland G, Richardson L, et al. Control of norovirus outbreak on a pediatric oncology unit. Am J Infect Control 2015; 43:1066–9. 26164767 10.1016/j.ajic.2015.05.032 PMC4988230

[7] Henke-Gendo C, Harste G, Juergens-Saathoff B, Mattner F, Deppe H, Heim A New real-time PCR detects prolonged norovirus excretion in highly immunosuppressed patients and children. J Clin Microbiol 2009; 47:2855–62. 19625473 10.1128/JCM.00448-09 PMC2738087

[8] Saif MA, Bonney DK, Bigger B, et al. Chronic norovirus infection in pediatric hematopoietic stem cell transplant recipients: a cause of prolonged intestinal failure requiring intensive nutritional support. Pediatr Transplant 2011; 15:505–9. 21504523 10.1111/j.1399-3046.2011.01500.x

[9] Brown JR, Roy S, Shah D, et al. Norovirus transmission dynamics in a paediatric hospital using full genome sequences. Clin Infect Dis 2018. doi: 10.1093/cid/ciy438 PMC6321856 29800111

[10] Kundu S, Lockwood J, Depledge DP, et al. Next-generation whole genome sequencing identifies the direction of norovirus transmission in linked patients. Clin Infect Dis 2013; 57:407–14. 23645848 10.1093/cid/cit287 PMC3703108

[11] Cotten M, Koopmans M Next-generation sequencing and norovirus. Future Virol 2016; 11:719–22. 28757893 10.2217/fvl-2016-0099

[12] Greninger AL, Zerr DM, Qin X, et al. Rapid metagenomic next-generation sequencing during an investigation of hospital-acquired human parainfluenza virus 3 infections. J Clin Microbiol 2017; 55:177–82. 27795347 10.1128/JCM.01881-16 PMC5228228

[13] Greninger AL, Waghmare A, Adler A, et al. Rule-out outbreak: 24-hour metagenomic next-generation sequencing for characterizing respiratory virus source for infection prevention. J Pediatric Infect Dis Soc 2017; 6:168–72. 28379561 10.1093/jpids/pix019 PMC5907853

[14] Kothari A, Burgess MJ, Crescencio JCR, et al. The role of next generation sequencing in infection prevention in human parainfluenza virus 3 infections in immunocompromised patients. J Clin Virol 2017; 92:53–5. 28531552 10.1016/j.jcv.2017.05.010 PMC5521260

[15] MacCannell T, Umscheid CA, Agarwal RK, Lee I, Kuntz G, Stevenson KB; Healthcare Infection Control Practices Advisory Committee (HICPAC). Guideline for the Prevention and Control of Norovirus Gastroenteritis Outbreaks in Healthcare Settings. Available at: https://www.cdc.gov/infectioncontrol/guidelines/norovirus/index.html Accessed 13 August 2018.

[16] Goya S, Valinotto LE, Tittarelli E, et al. An optimized methodology for whole genome sequencing of RNA respiratory viruses from nasopharyngeal aspirates. PLOS One 2018; 13:e0199714. 29940028 10.1371/journal.pone.0199714 PMC6016902

[17] Bankevich A, Nurk S, Antipov D, et al. SPAdes: a new genome assembly algorithm and its applications to single-cell sequencing. J Comput Biol 2012; 19:455–77. 22506599 10.1089/cmb.2012.0021 PMC3342519

[18] Martin M Cutadapt removes adapter sequences from high-throughput sequencing reads. EMBnet J 2011; 17:10–2.

[19] Kroneman A, Vennema H, Deforche K, et al. An automated genotyping tool for enteroviruses and noroviruses. J Clin Virol 2011; 51:121–5. 21514213 10.1016/j.jcv.2011.03.006

[20] Matsushima Y, Shimizu T, Ishikawa M, et al. Complete genome sequence of a recombinant GII.P16-GII.4 norovirus detected in Kawasaki City, Japan, in 2016. Genome Announc 2016; 4:e01099–16. 10.1128/genomeA.01099-16 PMC5054331 27795262

[21] Kearse M, Moir R, Wilson A, et al. Geneious Basic: an integrated and extendable desktop software platform for the organization and analysis of sequence data. Bioinformatics 2012; 28:1647–9. 22543367 10.1093/bioinformatics/bts199 PMC3371832

[22] Katoh K, Standley DM MAFFT multiple sequence alignment software version 7: improvements in performance and usability. Mol Biol Evol 2013; 30:772–80. 23329690 10.1093/molbev/mst010 PMC3603318

[23] Huelsenbeck JP, Ronquist F MRBAYES: Bayesian inference of phylogenetic trees. Bioinformatics 2001; 17:754–5. 11524383 10.1093/bioinformatics/17.8.754

[24] Debbink K, Donaldson EF, Lindesmith LC, Baric RS Genetic mapping of a highly variable norovirus GII.4 blockade epitope: potential role in escape from human herd immunity. J Virol 2012; 86:1214–26. 22090110 10.1128/JVI.06189-11 PMC3255819

[25] Parra GI, Squires RB, Karangwa CK, et al. Static and evolving norovirus genotypes: implications for epidemiology and immunity. PLOS Pathog 2017; 13:e1006136. 28103318 10.1371/journal.ppat.1006136 PMC5283768

[26] Norovirus, US Trends and Outbreaks, Centers for Disease Control and Prevention. Burden of Norovirus Illness in the US. Available at: https://www.cdc.gov/norovirus/trends-outbreaks/burden-US.html Accessed 2 November 2018.

[27] Xue KS, Stevens-Ayers T, Campbell AP, et al. Parallel evolution of influenza across multiple spatiotemporal scales. Elife 2017; 6:e26875. 10.7554/eLife.26875 PMC5487208 28653624

[28] Quick J, Loman NJ, Duraffour S, et al. Real-time, portable genome sequencing for Ebola surveillance. Nature 2016; 530:228–32. 26840485 10.1038/nature16996 PMC4817224

[29] Gardy JL, Loman NJ Towards a genomics-informed, real-time, global pathogen surveillance system. Nat Rev Genet 2018; 19:9–20. 29129921 10.1038/nrg.2017.88 PMC7097748

[30] Brown JR, Roy S, Ruis C, et al. Norovirus whole-genome sequencing by SureSelect target enrichment: a robust and sensitive method. J Clin Microbiol 2016; 54:2530–7. 27487952 10.1128/JCM.01052-16 PMC5035417

[31] Wylie TN, Wylie KM, Herter BN, Storch GA Enhanced virome sequencing through solution-based capture enrichment. Genome Res 2015. 10.1101/gr.191049.115 PMC4665012 26395152

[32] Zhou Y, Wylie KM, El Feghaly RE, et al. Metagenomic approach for identification of the pathogens associated with diarrhea in stool specimens. J Clin Microbiol 2016; 54:368–75. 26637379 10.1128/JCM.01965-15 PMC4733167

[33] Petrignani M, Verhoef L, de Graaf M, Richardus JH, Koopmans M Chronic sequelae and severe complications of norovirus infection: A systematic review of literature. J Clin Virol 2018; 105:1–10. 29804008 10.1016/j.jcv.2018.05.004

