# Supplementary figures and images for: Prospective, Real-time Metagenomic Sequencing During Norovirus Outbreak Reveals Discrete Transmission Clusters
Source: Clin Infect Dis. 2018 Dec 4;69(6):941–8. doi: 10.1093/cid/ciy1020 (PMC6735836; doi:10.1093/cid/ciy1020)

**A**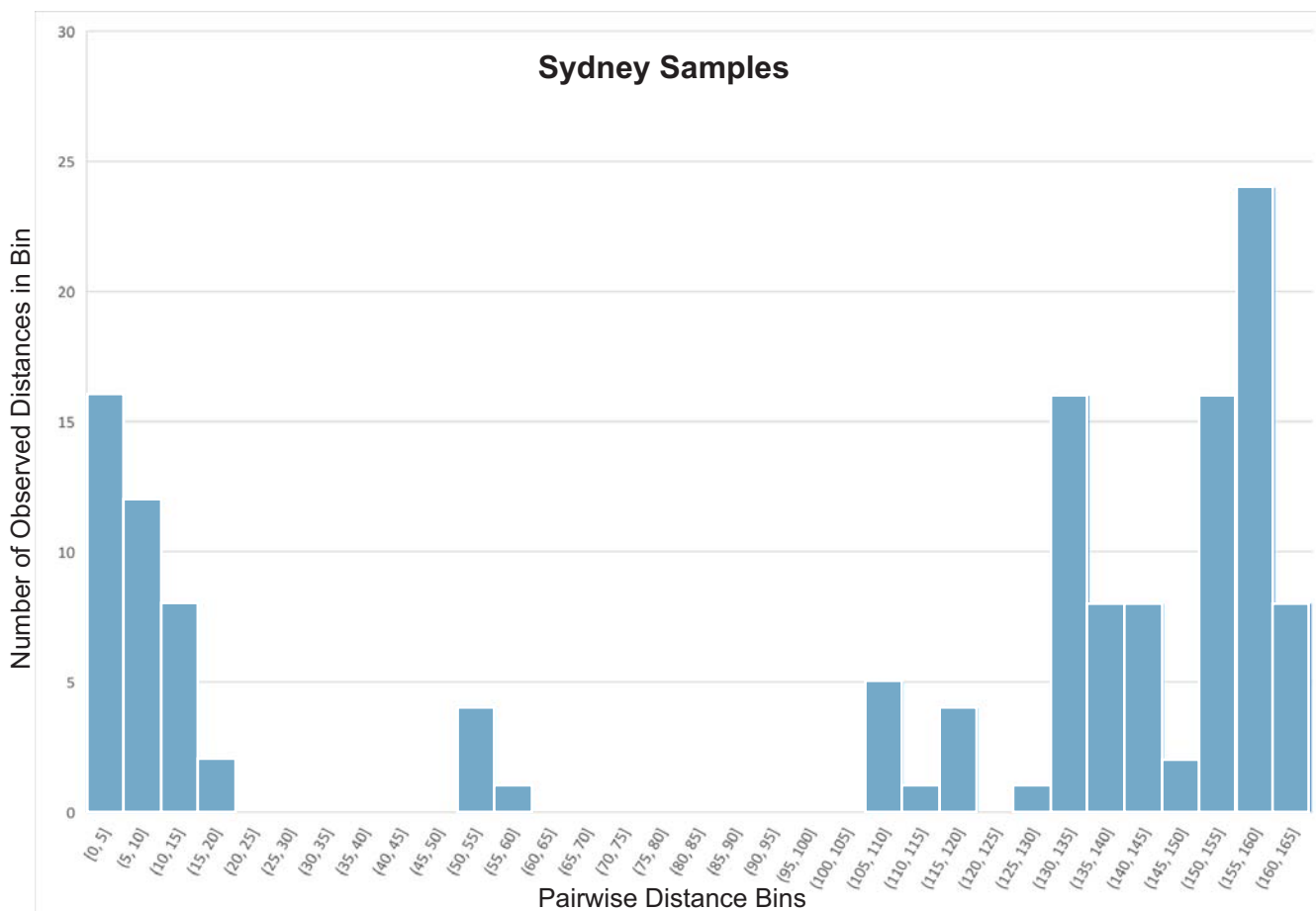**B**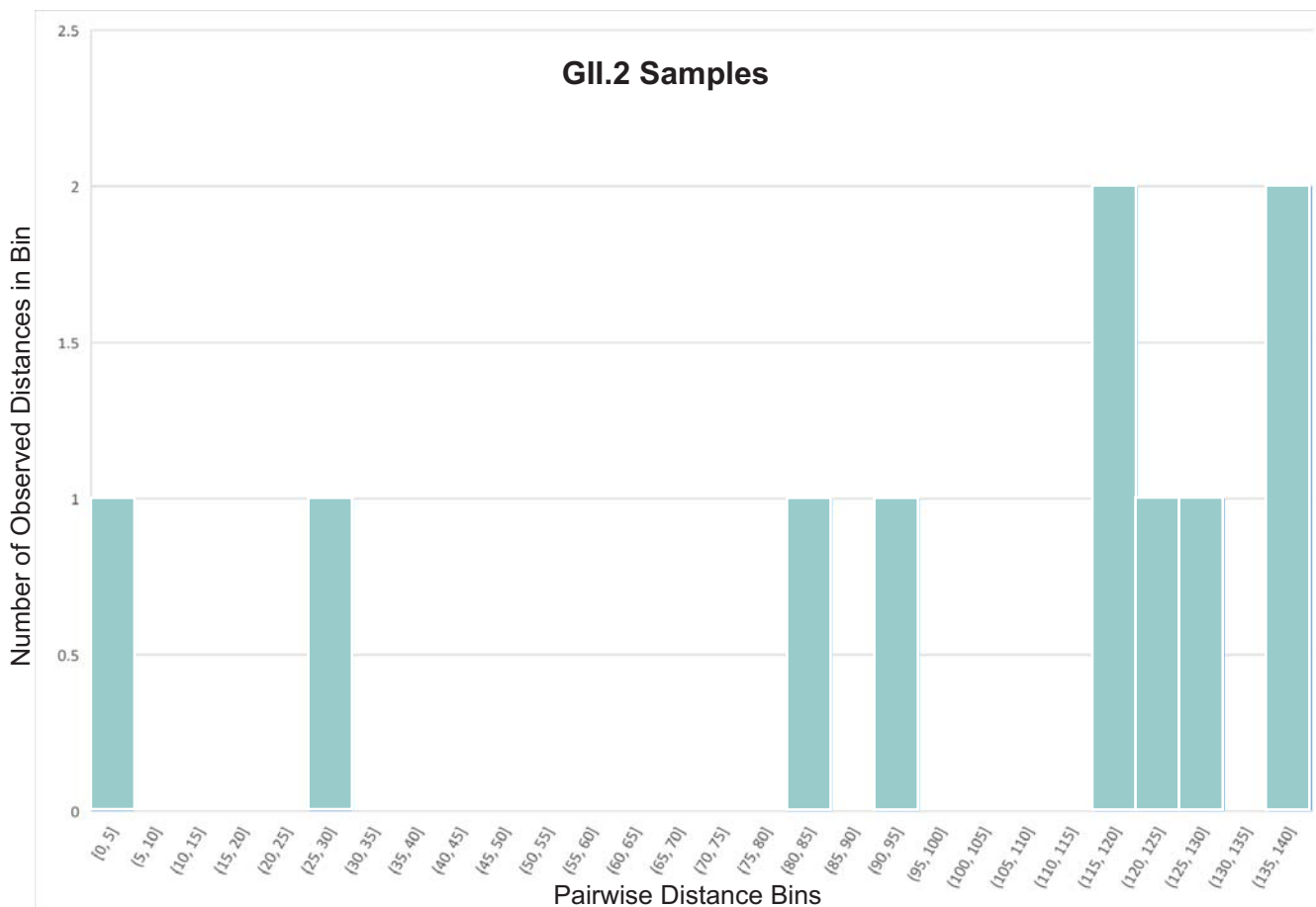

Supplement: ciy1020_suppl_Supplementary_Figure_S1 [file ciy1020_suppl_supplementary_figure_s1.pdf]

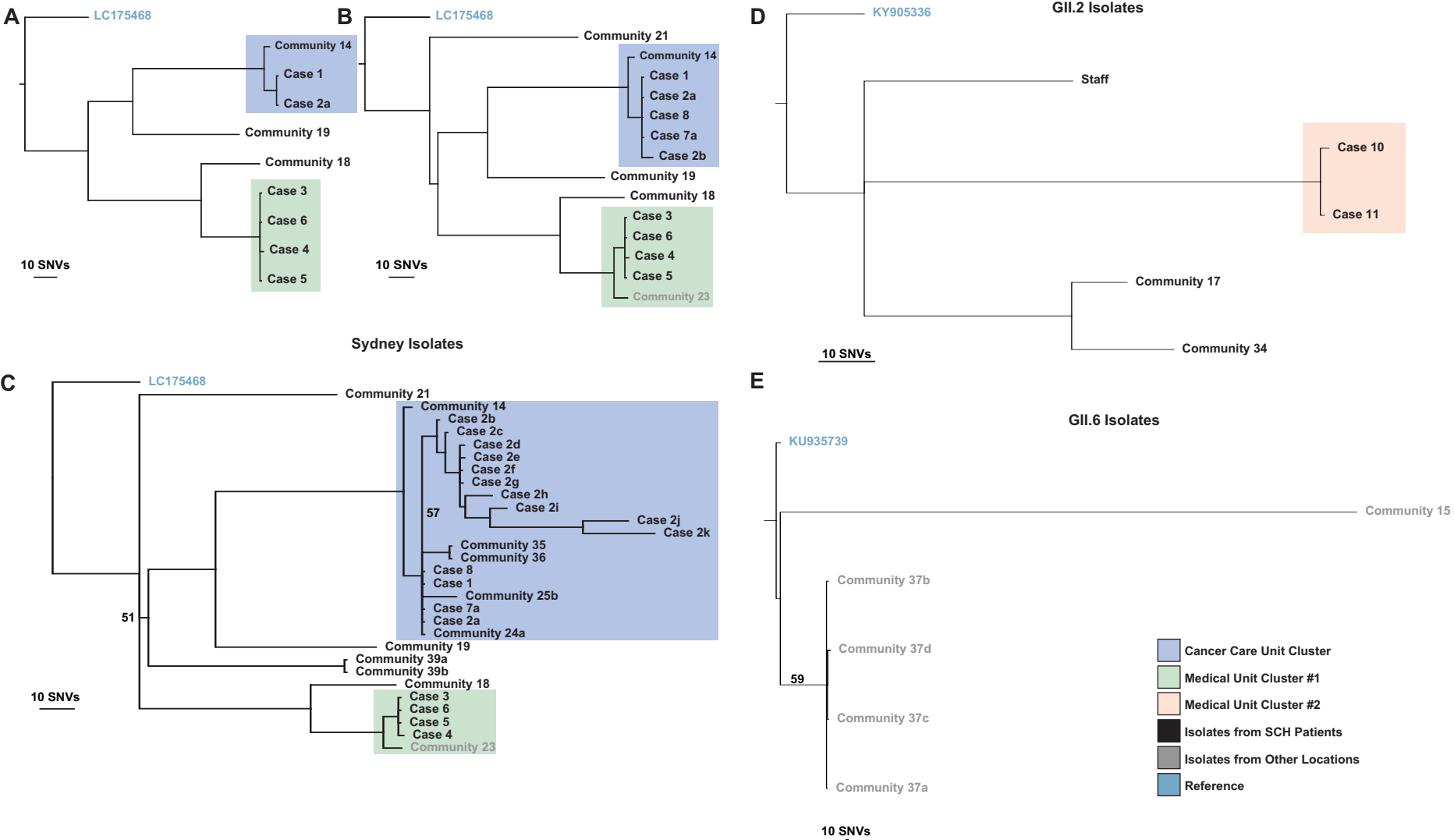

Supplement: ciy1020_suppl_Supplementary_Figure_S2 [file ciy1020_suppl_supplementary_figure_s2.pdf]

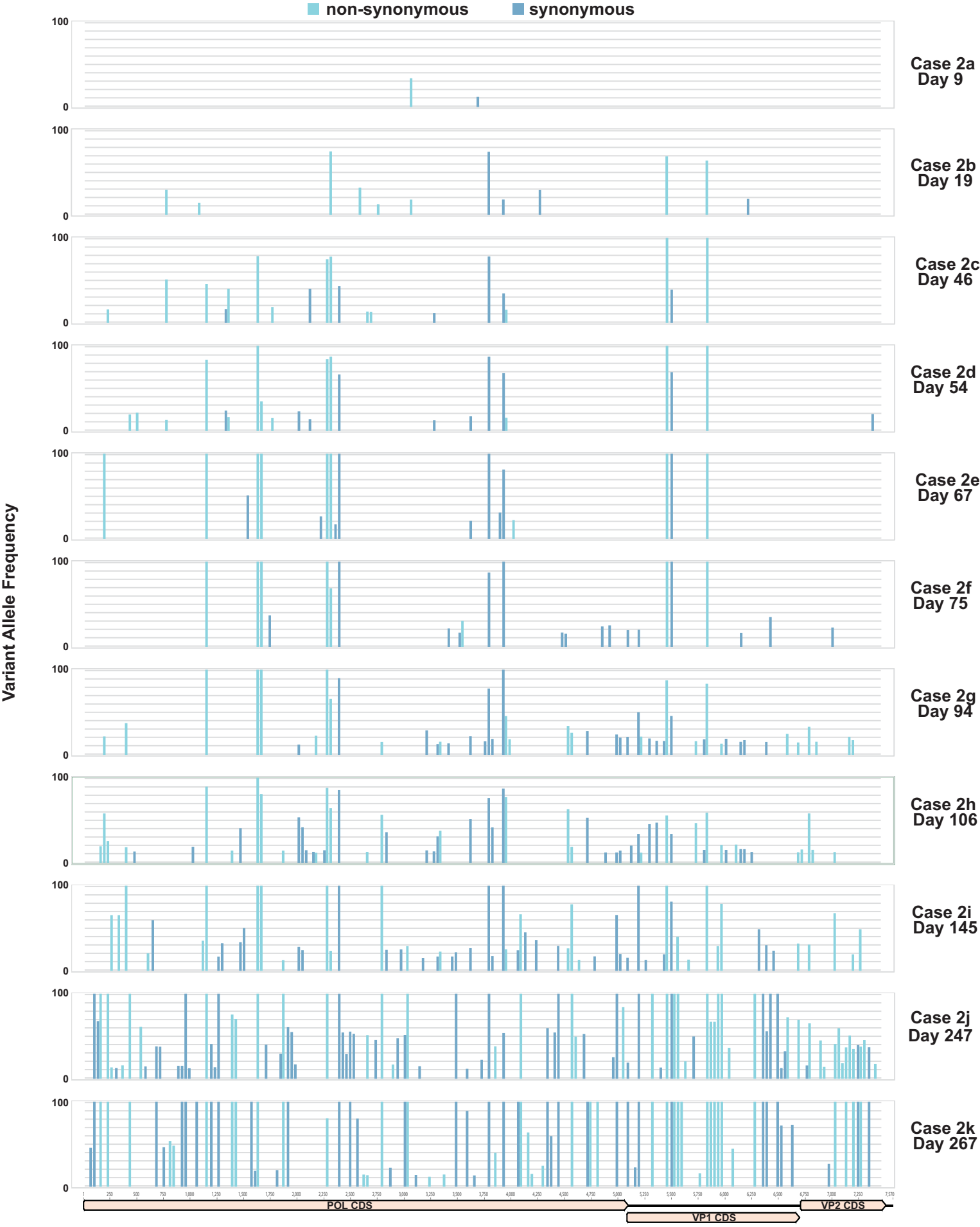

Supplement: ciy1020_suppl_Supplementary_Figure_S3 [file ciy1020_suppl_supplementary_figure_s3.pdf]

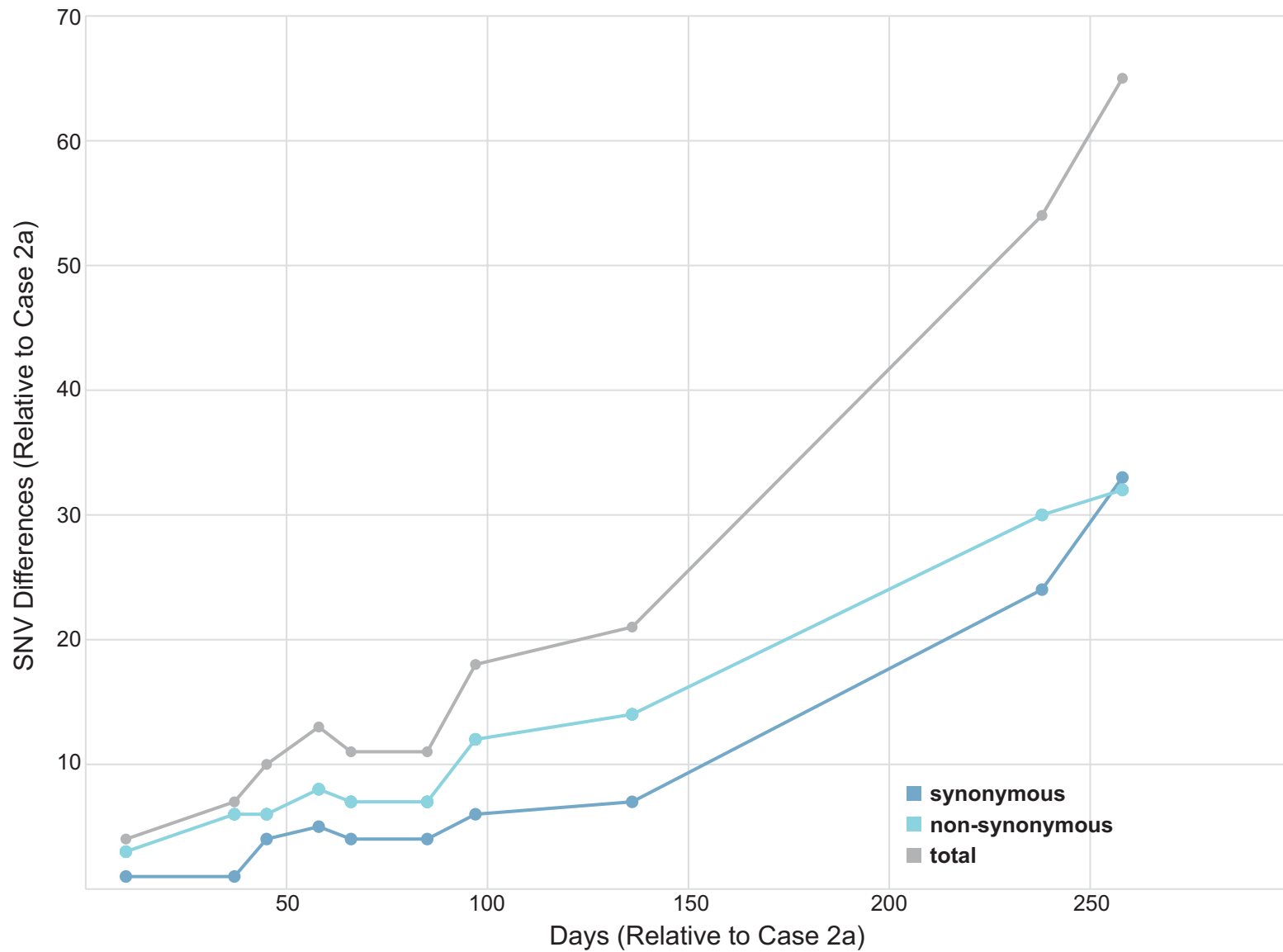

Supplement: ciy1020_suppl_Supplementary_Figure_S4 [file ciy1020_suppl_supplementary_figure_s4.pdf]
