# Supplementary material for: Prospective, Real-time Metagenomic Sequencing During Norovirus Outbreak Reveals Discrete Transmission Clusters
Source: Clin Infect Dis. 2018 Dec 4;69(6):941–8. doi: 10.1093/cid/ciy1020 (PMC6735836; doi:10.1093/cid/ciy1020)
Supplement: ciy1020_suppl_Supplementary_Notes [file ciy1020_suppl_supplementary_notes.docx]

**Supplementary Materials**

**Methods**

**Note S1**: Metadata for Hospital-Acquired and Staff Cases.

**Note S2**: Enteric Pathogen Co-Infection among Hospital-Acquired Norovirus Cases.

**Note S3**: dN/dS Analysis for other Chronically-Infected, Immunocompromised Hosts.

**Table S1**: Data for samples sequenced in this study. Columns are: GenBank Accession, Sample Name, Day of Sequencing Attempt, Cycle Time, Genotype assigned by Online Genotyping Tool, Samples with Consensus Sequences, Sample Cluster, Hospital-Acquired Cases, Total Number of Reads Generated, Number of Reads mapping to Corresponding Reference, Average Coverage, Percent of Consensus with greater than 0x Coverage, Percent of Consensus with greater than 10x Coverage, Number of Consensus Loci with 0x Coverage, Length of Consensus, First Base of Consensus relative to Reference, Last Base of Consensus relative to Reference, Number of Ns in Consensus, Number of Bases where Geneious called an Ambiguous Base and We Manually assigned a Non-Ambiguous Base, Number of Bases where Geneious called a Non-Ambiguous Base and We Manually assigned a Different Non-Ambiguous Base, Percent of Total Reads that were Norovirus, Percent of Total Reads that were Viral, Percent of Total Reads that were Bacterial, Percent of Total Reads that were Human.

**Table S2**: Pairwise SNV distance tables for all sequenced samples.

**Table S3**: Allele frequencies for all variant alleles observed in the samples from the Case 2 patient relative to Case 2a.

**Table S4**: Samples from immunocompromised patients including the Case 2 patient with number of total, synonymous, and non-synonymous changes relative to the first sample from each patient for the entire genome, ORF2, and the P2 subdomain; dN/dS valves for ORF2 and the P2 subdomain; and evolutionary rate values for ORF2.

**Table S5**: Norovirus RT-PCR primers and probes.

**Figure S1**: Histogram of pairwise distances between sequences. A) Pairwise distances between Sydney genotype sequences. B) Pairwise distances between GII.2 genotype sequences. Note that for both A and B pairwise distances were calculated for only one sample (the first one collected) per individual.

**Figure S2**: A) Phylogenetic tree for all Sydney samples sequenced on Day 9. B) Phylogenetic tree for all Sydney samples sequenced on Day 9 or Day 26. C) Phylogenetic tree for all sequenced Sydney samples. D) Phylogenetic tree for all sequenced GII.2 samples. E) Phylogenetic tree for all sequenced GII.6 samples.

**Figure S3:** Minor variants and consensus sequence changes for the norovirus from Case 2. Each rectangle represents the genome of a sample with genomic position on the X axis. The consensus sequence of Case 2a is used as a reference. Vertical bars represent the variant allele frequency (VAF) (each gray line denotes 10%) at a particular site with light blue bars representing non-synonymous changes and dark blue bars representing synonymous changes. Genomic position of the three norovirus ORFs is given at the bottom of the figure.

**Figure S4**: Consensus sequence changes in samples from Case 2 patient over time.

**Supplementary Methods**

*Infection Control measures*

All patients with gastroenteritis were placed in contact enteric precautions for the duration of the outbreak. Within 24 hours of detection of the outbreak, an initial communication was sent to providers describing the outbreak and providing recommendations aimed at reducing transmission and identifying additional cases. A subsequent communication was distributed to provide norovirus prevention education house-wide. Throughout the outbreak, there was heightened emphasis on hand hygiene practices (direct hand hygiene observations are standard), isolating symptomatic patients, and testing patients with health care-associated gastroenteritis. Staff were also frequently reminded what they should do if they developed symptoms of gastrointestinal illness. Occupational health developed employee stool specimen kits which contained a laboratory requisition, specimen container, spoon, and gloves. The kits were distributed to the units and staff were encouraged to submit stool samples if they developed GI symptoms. Environmental surfaces throughout the hospital were regularly cleaned with bleach during the outbreak.

*Norovirus testing*

Stool samples of symptomatic patients from SCH and other facilities within the University of Washington (UW) medical system were tested for norovirus at the UW clinical virology laboratory using a laboratory-developed quantitative real-time reverse transcription PCR assay that detects genogroups I and II [1]. However, once the outbreak was identified, in addition to the testing at UW, samples from suspected hospital-acquired cases were tested at SCH using the GI FilmArray assay (BioFire, Salt Lake City, UT), which detects 22 targets and has a shorter turnaround time [2]. Follow-up norovirus testing in patients involved in the outbreak was performed at the discretion of the clinical teams and was not part of the outbreak investigation.

*Stool sample preparation for norovirus RT-PCR at University of Washington*

Stool specimens were added to 1 mL of STAR buffer (Roche) using disposable loops or 100 uL of liquid sample was added to the buffer. For rectal swab samples, the swab was placed in the tube. Samples were vortexed for 10-15 seconds, the swab was removed, and the tubes were centrifuged at 8000 rpm for 5 minutes.

*RNA extraction*

Twenty uL of supernatant plus 180 uL HBSS were loaded into a MagNA Pure sample cartridge and total nucleic acids were extracted on the MagNA Pure LC instrument (Roche) using the MagNA Pure LC Total NA Isolation Kit-High Performance (Roche), eluting in 200 uL of buffer. A 130 base RNA transcript (EXO) was added to the lysis buffer to monitor for RNA extraction efficiency and the presence of amplification inhibitors.

*Norovirus RT-qPCR assay at University of Washington*

Real-time PCR primers and probes were designed to amplify a 90-100 base region of the norovirus ORF-1/ORF-2 junction, the most conserved region of norovirus [3], using sequences from human norovirus genogroups I and II. To cover the sequence diversity and limit degeneracy, the norovirus I RT-PCR assay contains 4 forward primers, 2 probes, and 1 reverse primer and the norovirus II RT-PCR assay contains 6 forward primers, 2 probes and 3 reverse primers (Supplementary Table S5). All TaqMan probes were labeled on the 5’ end with FAM and on the 3’ end with BHQ1. Primers and a VIC-labeled probe for amplification of EXO were included in each norovirus RT-PCR assay [4].

RT-PCR reactions were performed using the UltraSense One-Step Quantitative RT-PCR System (ThermoFisher). The norovirus I assay contained 500 nM each norovirus I primer and 100 nM each probe. The norovirus II assay contained 380 nM each norovirus II primer and 100 nM each probe. Both assays contained 100 nM EXO forward primer, 200 nM EXO reverse primer and 65 nM EXO probe. Ten uL of extracted sample was amplified. Thermocycling conditions were 50^o^C for 15 minutes, 95^o^C for 2 minutes, and 40 cycles of 95^o^C for 15 seconds and 60^o^C for 1 minute. Positive control and negative controls were included and co-processed with the samples.

*Community-Acquired Case Sample Selection*

All positive samples resulted at the UW clinical virology lab from Day -7 until Day 40 with a cycle time of 32.1 or less were subjected to mNGS. We also sequenced samples collected between Day 41 and Day 112 that came from patients for which a we had more than one sample and which had a cycle time of 31.1 or less. Finally, we sequenced three additional samples (collected on days 145, 247, and 267) from Case 2, who was chronically infected with the virus.

*mNGS Library Generation and Sequencing*

mNGS sequencing libraries were prepared as described previously [5,6]. Briefly, 20uL of stool suspension was mixed in 180uL of HBSS, bead beaten, and RNA was extracted on a MagnaPure 2.0 instrument (Roche) and eluted in 100uL water. 20uL of RNA was treated with Turbo DNAse (Thermo Fisher) and ds-cDNA was synthesized using random hexamers and SuperScript III reverse transcriptase (Thermo Fisher) and Sequenase 2.0 (Thermo Fisher) followed by cleaning using a Zymo DNA Clean and Concentrator-5 (Zymo Research). ds-cDNA was tagmented using third-reactions with the Nextera XT kit (Illumina) followed by 20 cycles of dual-indexed amplification [7]. Libraries were sequenced using 1x192 runs on an Illumina MiSeq. Reads were quality and adapter trimmed using cutadapt and analyzed as below [8].

*Consensus Generation.*

Raw reads were trimmed and then mapped to the corresponding reference genome in Geneious. The consensus genomes as called by Geneious were then manually reviewed. In most cases, we accepted the genomes as called by Geneious though the following situations should be noted:

1) The beginning and end of a consensus sequence were determined by where coverage dropped to 0. The first and last bases of the consensus for each sample relative to the corresponding reference are listed in Supplementary Table S1.

2) If there was a coverage gap for a sequence (a region not at either end of the sequence where coverage went to 0), all bases within the gap in the consensus were assigned a value of N. The one exception to this was for the samples from the immunocompromised patient. For these consensus sequences, we used sequences from the other samples to impute the value of bases that had 0x coverage. The number of bases with 0x coverage in each consensus sequence is listed in Supplementary Table S1.

3) All consensus sequences had some loci where Geneious called an ambiguous base (such as R, Y, S, W, etc). Most commonly, these ambiguous base calls occurred in groups and corresponded to reads that began or ended with a series of nucleotide mismatches relative to the reference. If there was at least one read that spanned this region and carried the reference sequence, we assigned these ambiguous bases the nucleotides of the reference sequence. If all reads had a series of nucleotide mismatches relative to the reference, we assigned these bases a value of N in the consensus.

4) For a small number of bases, Geneious called a non-ambiguous base (A, C, G, T) that we felt was erroneous based on our review of the reads. In these cases, we manually corrected the base.

*Sequence Clusters*

The selection of 10 SNP differences as the cut-off value for sequencing clustering was based on previous estimates of the norovirus mutation rate and on the observed distribution of pairwise differences among our samples.

The majority of our samples were of the Sydney genotype. The mutation rate for ORF2 for this genotype has been estimated to be 5.4 x 10^-3^, which converts to about 4 mutations/genome/month [9]. The mutation rate of the rest of the genome is likely slower than this, so we estimated a mutation rate range of 2 – 4 mutations/genome/month. We wanted to cluster together any samples that had a common ancestor within the current norovirus peak season. As the outbreak began in early January, a 6 week period prior to its beginning would cover most of the winter season when norovirus cases sharply increase [10]. This mutation rate range and this timeframe gave us a cut-off range of about 6 – 12 SNPs. We then looked at the distribution of pairwise differences for our samples to see if there were any clear separations among these distances. Supplementary Figure S1 shows these distances for the Sydney and GII2 samples (only one sample per individual was considered). With the Sydney samples, distances are either less than or equal to 15 or greater than 50. A clustering cut-off of 10 placed together in a cluster all samples that had a distance of 15 or less.

If we used a cut-off of 5 SNPs instead of 10, the “core” hospital-acquired clusters would remain intact: Cancer Care Unit Cluster – Case 1, Case 2, Case 7, Case 8, Community 24, Medical Unit Cluster #1 – Case 3, Case 4, Case 5, Case 6, Medical Unit Cluster #2 – Case 10, Case 11. The additional community-acquired cases, though, would not have grouped with the Cancer Care Unit Cluster and Medical Unit Cluster #1. Since we were interested in connections between the hospital-acquired cases and norovirus strains circulating in the community, we chose the more liberal cut-off of 10.

*Minor variant calling*

Contigs for each sample from Case 2 were mapped to the corresponding reference sequence using Geneious. A mega file was generated from this alignment and a python script was used to screen this file for minor variants for each sequence we generated. Specifically, this script returned a locus if it 1) had a coverage depth of at least 10, 2) displayed a mixture of at least 2 different alleles at that locus with the minor allele frequency equal to 10% or greater, and 3) was not bordered by sequence with differences relative to the reference exceeding a certain cutoff. All putative minor variants returned by this script were then manually reviewed and about half were excluded as being likely secondary to sequencing artifact.

**Supplementary Note S1. Hospital-Acquired and Staff Cases Metadata**

Hospital-acquired norovirus case patients on the medical unit were a median of 0.8 years (range 0.3-1.6 years) old and all patients had underlying chronic conditions (chronic lung disease = 7 and rheumatic disease = 1). Patients on the cancer care unit were a median of 1.6 years (range 1-20 years) old. Four patients were undergoing treatment for cancer and one patient was 2 months post-HCT.

Staff cases included 46 nurses or nursing assistants, 3 physicians, and 33 non-clinical staff; the roles of the remaining 4 staff members were not recorded.

**Supplementary Note S2. Enteric Pathogen Co-Infection Among Hospital-Acquired Norovirus Cases**

In addition to norovirus, 5 hospital-acquired case patients tested positive for other enteric pathogens. Of the 8 case patients on the medical unit, 4 (50%) tested positive for another enteric pathogen. Two of these patients were positive for *Clostridium difficile*, one patient was positive for rotavirus, and one patient was positive for both *Yersinia enterocolitica* and enteropathogenic *E. coli*. Of the 5 case patients on the cancer care unit, 1 (20%) tested positive for a second enteric pathogen (*C. difficile).*

**Supplementary Note S3. dN/dS Analysis for other Chronically Infected Immunocompromised Hosts**

To assess whether these dN/dS and mutation rate values are typical for chronically-infected, immunocompromised patients, we calculated these same parameters for 13 additional patients infected with GII.4 norovirus (Genbank accessions MF140633-MF140697, Supplementary Table S4) [11]. There were 2 – 10 samples available for each of these patients. The average dN/dS for ORF2 for all 14 immunocompromised patients was 1.950 (standard deviation 1.204). dN/dS could not be calculated in four comparisons as no synonymous changes were observed. For the P2 subdomain, the average dN/dS was 3.949 (standard deviation 2.12). dN/dS could not be calculated for 26 comparisons. Finally, the average mutation rate across all 14 patients for ORF2 was 0.018 substitutions/site/year (standard deviation 0.012).

**Supplementary References**

1. Kageyama T, Kojima S, Shinohara M, et al. Broadly reactive and highly sensitive assay for Norwalk-like viruses based on real-time quantitative reverse transcription-PCR. J Clin Microbiol **2003**; 41:1548–1557.

2. Cybulski RJ, Bateman AC, Bourassa L, et al. Clinical impact of a Multiplex Gastrointestinal PCR Panel in Patients with Acute Gastroenteritis. Clin Infect Dis **2018**;

3. Kageyama T, Kojima S, Shinohara M, et al. Broadly reactive and highly sensitive assay for Norwalk-like viruses based on real-time quantitative reverse transcription-PCR. J Clin Microbiol **2003**; 41:1548–1557.

4. Limaye AP, Huang ML, Leisenring W, Stensland L, Corey L, Boeckh M. Cytomegalovirus (CMV) DNA load in plasma for the diagnosis of CMV disease before engraftment in hematopoietic stem-cell transplant recipients. J Infect Dis **2001**; 183:377–382.

5. Greninger AL, Zerr DM, Qin X, et al. Rapid Metagenomic Next-Generation Sequencing during an Investigation of Hospital-Acquired Human Parainfluenza Virus 3 Infections. J Clin Microbiol **2017**; 55:177–182.

6. Goya S, Valinotto LE, Tittarelli E, et al. An optimized methodology for whole genome sequencing of RNA respiratory viruses from nasopharyngeal aspirates. PLoS ONE **2018**; 13:e0199714.

7. Iketani S, Shean RC, Ferren M, et al. Viral Entry Properties Required for Fitness in Humans Are Lost through Rapid Genomic Change during Viral Isolation. MBio **2018**; 9.

8. Martin M. Cutadapt removes adapter sequences from high-throughput sequencing reads. EMBnet.journal **2011**; 17:10.

9. Parra GI, Squires RB, Karangwa CK, et al. Static and Evolving Norovirus Genotypes: Implications for Epidemiology and Immunity. PLoS Pathog **2017**; 13:e1006136.

10. Norovirus | U.S. Trends and Outbreaks | CDC. 2018. Available at: https://www.cdc.gov/norovirus/trends-outbreaks/burden-US.html. Accessed 2 Norovirusember 2018.

11. van Beek J, de Graaf M, Smits S, et al. Whole-Genome Next-Generation Sequencing to Study Within-Host Evolution of Norovirus (Norovirus) Among Immunocompromised Patients With Chronic Norovirus Infection. J Infect Dis **2017**; 216:1513–1524.
